# Factors associated with low adherence to medication among patients with type 2 diabetes at different healthcare facilities in southern Bangladesh

**DOI:** 10.1080/16549716.2021.1872895

**Published:** 2021-01-21

**Authors:** Adnan Mannan, Md. Mahbub Hasan, Farhana Akter, Md. Mashud Rana, Nowshad Asgar Chowdhury, Lal B. Rawal, Tuhin Biswas

**Affiliations:** a Department of Genetic Engineering & Biotechnology, Faculty of Biological Sciences, University of Chittagong, Chattogram, Bangladesh; b Department of Endocrinology, Chittagong Medical College, Chattogram, Bangladesh; c Department of Pharmacology and Therapeutics, Chittagong Medical College, Chattogram, Bangladesh; d Office of the Deputy Director, Chattogram Diabetic General Hospital, Chattogram, Bangladesh; e School of Health, Medical and Applied Sciences, Central Queensland University, Sydney Campus, Australia; f ARC Centre of Excellence for Children and Families over the Life Course, The University of Queensland, Brisbane, Australia; g Institute for Social Science Research, The University of Queensland, Long Pocket Precinct, Brisbane, Australia

**Keywords:** Jennifer Stewart Williams, Umeå University, Sweden, Medication adherence, type 2 diabetes, diabetic ulcer, Chittagong, Bangladesh

## Abstract

**Background**: Diabetic individuals must adhere to their medications to control their glucose levels and prevent diabetes-related complications. However, there is limited evidence of medication adherence in patients with type 2 diabetes in Bangladesh.

**Objectives**: We assessed the level of adherence and factors associated with low adherence to anti-diabetic medication among patients with type 2 diabetes at different health facilities in southern Bangladesh.

**Methods**: This cross-sectional study included 2,070 patients with type 2 diabetes who presented at five health facilities in the Chittagong Division between November 2018 and June 2019. We assessed medication adherence using a self-reported, structured, eight-item questionnaire and performed multiple logistic regression to investigate the factors associated with low medication adherence.

**Results**: The overall prevalence of low medication adherence was 46.3% (95% CI: 41.4–55.8%) of our study population. Multiple logistic regression analysis revealed that males (OR: 1.37; 95% CI: 1.13–1.67), those with a family income of < 233 USD (OR: 1.54, 95% CI: 1.17–2.03), and those with a diabetic ulcer (OR: 1.42, 95% CI: 1.04–1.94) showed low adherence. Diabetic ulcers, retinopathy, and obesity were relatively more elevated among diabetic patients with low medication adherence.

**Conclusion**: Low medication adherence among patients with type 2 diabetes in southern Bangladesh is a key public health challenge. Factors such as male sex, low annual family income, and diabetic ulcers were associated with low medication adherence. Patient counseling and awareness programs may enhance medication adherence among people with type 2 diabetes. Our findings will help physicians and public health workers to develop targeted strategies to increase awareness of the same among their patients.

## Background

The prevalence of diabetes mellitus (DM) is increasing globally. There are four main types of diabetes - type 1, type 2,  gestational and pre-diabetes. All are complex and serious conditions. Globally, type 2 diabetes is the most prevalent form and the subject of this study. The overall morbidity and mortality rates of diabetes are higher in most low- and middle-income countries as compared with those of high-income countries [1]. Currently, Southeast Asia leads the diabetes frequency tally with 50% of global diabetes cases; this number is projected to increase to 70.6% by 2035 [2]. According to the International Diabetes Federation, approximately 8.4 million people were diagnosed with diabetes in Bangladesh in 2011, and a similar number of pre-diabetes cases were found in the same year. These figures are projected to increase by two-fold over the next two decades [3]. The results of a nationwide survey in 2011–12 established that the comprehensive, age-standardized prevalence of diabetes and pre-diabetes in Bangladesh was 9.7% and 22.4%, respectively, and the age-standardized prevalence was almost double in urban residents as compared with rural residents (15.2% vs. 8.3%) [4]. The prevalence of non-communicable diseases, including diabetes, is increasing in Bangladesh [5,6]. This poses a serious threat to the health system of Bangladesh, which is not yet sufficiently well prepared to effectively prevent and manage non-infectious conditions [3,4,7].

Diabetes accounts for a considerable number of premature morbidity and mortality rates [7,8]. A previous study in Bangladesh reported a high frequency of complications within cohorts who were newly diagnosed with type 2 diabetes [9]. Prevention of diabetes involves effective and successful glycemic control as well as the appropriate and timely use of medications, which may continue life-long [10]. Medication adherence has been defined as the ‘active, voluntary, and collaborative involvement of the patient in a mutually acceptable course of behavior to produce a therapeutic result’ [11]. Unfortunately, more than half the patients diagnosed with chronic diseases become and remain non-adherent to their prescribed treatment measures [12]. Adherence to medication among diabetic patients is poor in several countries, including Jamaica (30%) and Mexico (54%) [13,14]. Moreover, 25% of non-adherent adults with type 2 diabetes report poor glycemic control [15,16].

According to many previous studies, poor patient-provider relationships, prolonged intervals while delivering diabetes education, side effects of medications, and pill burdens are common risk factors for non-adherence to diabetes treatments [17]. However, all these studies were conducted in developed countries [4,7], whereas in low- and middle-income nations, such as Bangladesh, this issue and its manifestations remain largely unexplored [8]. To shed more light on this subject, we aimed to determine the levels of medication adherence in patients with type 2 diabetes from five health facilities in southern Bangladesh and analyze the factors associated with poor adherence.

## Methods

### Study design, setting and participants

This cross-sectional study was conducted between November 2018 and June 2019 among patients attending five health facilities in Chittagong, namely: Chittagong Medical College Hospital, Chattogram Diabetic General Hospital, Center for Specialized Care & Research (CSCR), Popular Diagnostics and Max Hospital. All five facilities are situated in Chattogram, the second-largest city of Bangladesh, and have a capacity of more than 100 beds. They serve around twomillion residents in Chattogram city and adjacent districts.

We included adults who were: diagnosed with type 2 diabetes mellitus as per the WHO criteria, taking oral medication for diabetes, registered at a hospital, referred by their attending physician, and residents of Chittagong city. According to our sample size calculation we required 1950 diabetes patients using our inclusion criteria. We excluded patients with other types of diabetes, those with type 1 diabetes, and those with serious illnesses that required hospitalization. Our sample size calculation and inclusion criteria allowed us to enroll 2070 diabetes patients in this study. We collected data from outpatient services at the five selected hospitals in a private setting.

### Ethical considerations

This study was approved by the Ethical Review Committee of Chittagong Medical College Hospital (CMC/PG/2019/57). It is a part of the project ‘Study on the molecular basis of the risk factors of diabetes and its association with the development of various comorbidities among diabetic patients in the southern part of Bangladesh’. We obtained written informed consent from all participants before the interview and explained the study objectives and procedures to them in their native language (Bengali).

### Data collection and variables

We administered a pre-tested, structured questionnaire to collect data. Five study physicians, two research officers, and fifteen research assistants were trained and involved in data collection and the principal and co-principal investigators carried out random cross-checks to ensure the quality of the data. The participants were interviewed face-to-face. Anthropometric measurements, i.e. weight, height, and body mass index (BMI), were measured using standardized protocols and calibrated equipment. Systolic and diastolic blood pressure was measured twice using digital monitors (Omron, SEM-1, Omron Corp., USA) and the patients were asked to rest for at least 10 minutes in the sitting position between readings. The average of the two readings was used for this analysis.

We collected and analyzed blood samples using standard protocols at the biochemistry laboratory to check glycated hemoglobin (HbA1c) levels. The glycemic status was considered controlled when the HbA1c was ≤ 7% and uncontrolled when the HbA1c was >7% according to the 2017 guidelines of the American Diabetes Association [18].

We assessed the medication adherence using a self-reported, structured, eight-item questionnaire that has been validated in different study settings [19,20] with the following cutoff scores: >2 = low adherence, 1 or 2 = medium adherence, 0 = high adherence [21]. The eight items in the questionnaire were: (1) medication frequency, (2) history, (3) deliberately missed doses, (4) cutting back without consultation, (5) forgetfulness while traveling, (6) stopping in an under-control situation, (7) inconvenience of taking medicine every day, and (8) difficulties in remembering to take the medicine. The questionnaire also requested information on socio-demographic characteristics, family history of diabetes, duration of diabetes, number of medications, self-reported comorbidities, and use of medication. We recorded the participants’ total duration of diabetes, duration of hypertension, and comorbidities using their responses in addition to reviewing their medical records and laboratory reports.

The questionnaires were translated by the research team using the standard forward-and-backward translation method suggested by the WHO and pre-tested in a small sample of 30 patients at a diabetes clinic in Chittagong. The questionnaire was reliable and showed good concurrent and predictive validity (93% sensitivity and 53% specificity) in low-income patients; it has been widely used as a screening tool during research in outpatient settings [22,23]. Because the prevalence of medium adherence was low in our study, we combined medium and high adherence as a single entity (“medium-to-high adherence”) in our analysis and compared as low adherence versus high or moderate adherence.

### Data analysis

The data were cleaned and analyzed using the SPSS 20.0 statistical software (IBM Corporation, Armonk, NY). Descriptive statistics were used to analyze the demographic characteristics of patients and their medication adherence scores. Categorical variables were expressed as percentages and frequencies, whereas mean and standard deviation (SD) was calculated for the continuous variables. Fisher’s exact and Chi-square tests were performed to find the group-wise association for categorical variables. Binary logistic regression was applied to test the presence of association with dichotomous outcomes: low adherence, medium-to-high adherence, and high adherence considered as a reference point. The independent variables for logistic regression were selected *a priori* from evidence in the literature and the statistical significance as per our bivariate analysis [17]. In the final model (Table 3), we included only variables that were significant in Tables 1 and 2 (gender, participants’ education, income, co-morbidities, and low consumption of fruits and vegetables).

**Table 1.** Socio-demographic characteristics of the study participants

	Total		High and Moderate MA		Low MA		
Variables	N	%	N	%	N	%	P-value^#^
**Gender**							
Male	1233	59.8	626	56.6	607	63.6	0.001*
Female	828	40.2	480	43.4	348	36.4	
**Age**							
Men age ± SD	50.6 ± 12.1		50.9 ± 12.2		50.4 ± 12		
<35 years	201	9.8	102	9.2	99	10.4	0.236
35–69 years	1725	83.7	923	83.5	802	84	
> 70 years	135	6.6	81	7.3	54	5.7	
**Marital status**							
Married	1846	89.6	993	89.8	853	89.3	0.731
Single	215	10.4	113	10.2	102	10.7	
**Education**							
Primary and below	823	39.9	417	37.7	406	42.5	0.015*
Completed secondary	550	26.7	289	26.1	261	27.3	
Completed higher secondary	302	14.7	184	16.6	118	12.4	
Completed higher education	386	18.7	216	19.5	170	17.8	
**Occupation**							
Housewife	1093	53	558	50.5	535	56	0.071
Business	243	11.8	142	12.8	101	10.6	
Service	395	19.2	218	19.7	177	18.5	
Others	330	16	188	17	142	14.9	
**Income**							
< 233 USD	578	28.5	286	26.2	292	31.2	0.002*
233–410 USD	627	30.9	320	29.3	307	32.8	
411–585 USD	397	19.6	230	21.1	167	17.8	
> 585 USD	426	21	255	23.4	171	18.2	

^#^Chi-square test unless otherwise stated * MA = Medication Adherence.

**Table 2.** Personal medical history and behavioral characteristics according to medication adherence

	Total		High and Moderate		Low		
Variables	N	%	N	%	N	%	P-value^#^
***Co-morbidities***							
Hypertension	1111	53.9	600	54.2	511	53.5	0.116
Heart diseases	431	20.9	226	20.4	205	21.5	0.736
Eye diseases	1155	56	566	51.2	589	61.7	< 0.001*
Kidney diseases	171	8.3	89	8	82	8.6	0.658
Neurological diseases	193	9.4	91	8.2	102	10.7	0.057
Diabetic ulcer	185	9	80	7.2	105	11	0.003*
Cancer	26	1.3	16	1.4	10	1	0.418
Asthma	240	11.6	115	10.4	125	13.1	0.058
TB	45	2.2	29	2.6	16	1.7	0.143
**Fasting blood sugar**							
Uncontrolled (>7 mmol/L)	1364	73.5	715	72.1	649	75.1	0.149
Controlled (≤7 mmol/L)	491	26.5	276	27.9	215	24.9	
**Body mass index**							
Underweight	34	1.6	17	1.5	17	1.8	0.57
Normal	855	41.5	455	41.1	400	41.9	
Overweight	845	41	467	42.2	378	39.6	
Obese	327	15.9	167	15.1	160	16.8	
**Behavioral characteristics**							
Tobacco use							
Current	550	26.7	283	25.6	267	28	0.289
Past	208	10.1	120	10.8	88	9.2	
Never	1303	63.2	703	63.6	600	62.8	
**Consumption of fruits and vegetables**							
≥3 times/day	101	4.9	41	3.7	60	6.3	0.007*
<3 times/day	1960	95.1	1065	96.3	895	93.7	

*Statistical significance at *P < 0.05; **P < 0.001; ^#^ Chi-square test unless otherwise stated.

**Table 3.** Factors associated with low medication adherence among diabetes patients

Factors	Unadjusted OR (95% CI)	P-value	Adjusted OR (95% CI)	P-value
**Gender**				
Male	1.34 (1.12–1.60)	0.001*	1.37(1.13–1.67)	0.002*
Female	Ref.		Ref.	
**Education**				
Primary and below	Ref.		Ref.	
Secondary	0.93(0.75–1.15)	0.495	1.04(0.82–1.3)	0.76
Higher secondary	0.66(0.5–0.86)	0.002*	0.79(0.59–1.05)	0.099
Higher education	0.81(0.63–1.03)	0.086	1.1(0.83–1.45)	0.527
**Income**				
< 233 USD	1.52(1.18–1.96)	0.001*	1.54(1.17–2.03)	0.002*
233–410 USD	1.43(1.12–1.84)	0.005*	1.43(1.1–1.84)	0.007*
411–585 USD	1.08(0.82–1.43)	0.575	1.08(0.81–1.43)	0.613
> 585 USD	Ref.		Ref.	
**Co-morbidities**				
Eye problems^a^	1.54(1.29–1.83)	<0.001	1.45(1.21–1.74)	P < 0.001
Diabetic ulcer^a^	1.58(1.17–2.15)	0.003*	1.42(1.04–1.94)	0.027*
**Consumption of fruits and vegetables**				
≥3 times/day	Ref.		Ref.	
<3 times/day	0.57(0.38–0.86)	0.008*	0.53(0.35–0.81)	0.003*

^a^No problems; *Statistical significance at P < 0.001.

## Results

We included 2070 patients in this study, nine of whom did not have complete information regarding medication adherence. Therefore, we finally analyzed 2061 patients. The socio-demographic characteristics of the participants according to medication adherence are summarized in Table 1. The mean age (±SD) of the participants was 50.6 (±12.1) years and more than 40% (40.2%) of them were female. A majority of the participants were married (89.6%) and educated up to the secondary school level (26.7%) (Table 1).

### Prevalence of medication adherence

**Figure 1.**
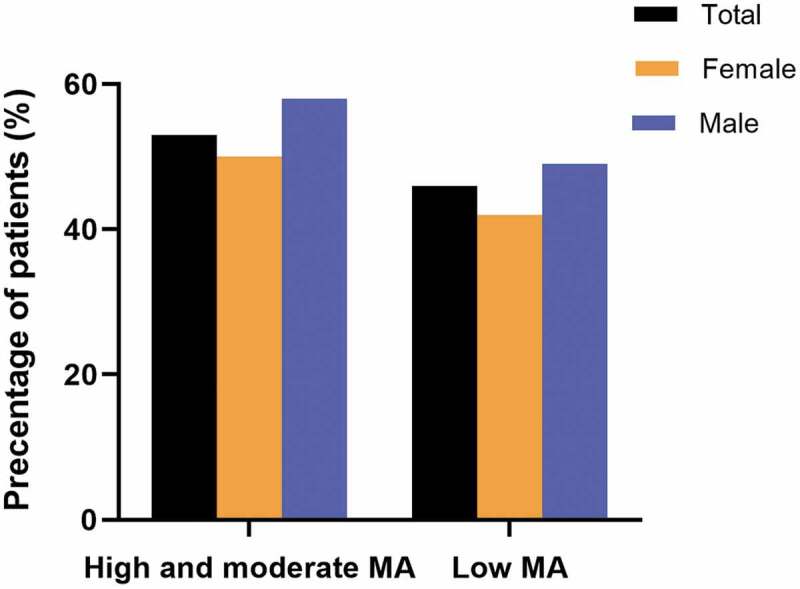
Medication adherence (MA) by gender

The overall prevalence of low adherence was 46.3% of participants (95% CI: 41.4–55.8%) and medium-to-high adherence was 53.7% (95% CI: 44.2–48.6%) (Figure 1). Prevalence of low adherence was higher among male subjects (49.2%, 95% CI: 46.4–52.0%) than female subjects (42.0%, 95% CI: 38.9–45.4%), which was statistically significant (p < 0.005) (Figure 1). Table 2 presents the personal medical history and behavioral characteristics of the patients according to their medication adherence and shows that comorbid conditions, such as heart diseases (21.5% vs. 20.4%), eye problems (61.7% vs. 52.2%), kidney diseases (8.6% vs 8.0%), neurological diseases (10.7% vs 8.2%), and diabetic ulcers (11.0% vs. 7.2%), were comparatively higher among diabetic patients having low adherence to medications. Additionally, we observed uncontrolled blood glucose (>7 mmol/L) and obesity among less-adherent patients.

### Factors responsible for low medication adherence

As per the adjusted bivariate logistic regression analyses, male participants had 1.4 times (OR: 1.37, 95% CI: 1.13–1.67) higher odds of low medication adherence as compared to their female counterparts (Table 3). Participants with family incomes of < 233 USD or 233–410 USD were 1.5 times (OR: 1.54, 95% CI: 1.17ךּ–2.03) and 1.4 times (OR: 1.43, 95% CI: 1.1–1.84) more likely to have low medication adherence, respectively, as compared to those with a family income of > 585 USD. In addition, participants who had diabetic ulcers (OR: 1.42, 95% CI: 1.04–1.94) and consumed lesser fruits and vegetables (< 3 times) per day (OR: 0.53, 95% CI: 0.35–0.81) showed higher chances of low medication adherence.

## Discussion

Our results showed that almost half of the participants with type 2 diabetes had low medication adherence. A study in Bangladesh measured medication adherence among patients with type 2 diabetes and reported that 20% of the participants were non-adherent to oral medication [24], which is lower than our findings. In contrast, Saleh et al. showed higher adherence in their study population, but this might be because they did not measure adherence using a standardized questionnaire. Our findings are in line with a report from India that used a standard medication-adherence tool and found 51.7% of their participants to have low adherence [25]. From these varied findings, it is clear that the lack of standard techniques to measure adherence, differences in sample populations, and the use of different definitions of glycemic control make it challenging to compare these studies.

Self-reported questionnaires that determine adherence are economical and easy to execute, but they usually overestimate adherence because patients tend to give socially acceptable responses. Moreover, recall bias among the participants regarding their medication-taking practices is entirely plausible [26]. These factors might be why we observed half of our participants having low adherence, in contrast to what has been observed qualitatively. Other adherence measurement tools, such as medication refill records, pill counts, and electronic monitors, might provide a less biased assessment of medication adherence; however, these are indirect tools and none of them can confirm the actual medicine consumption. There is also a chance that the patient might intentionally or unintentionally influence the result. In contrast, direct assessment tools, such as drug assays of blood and urine, can capture the appropriate picture and have fewer chances of being manipulated [27]. A previous systematic review reported that a majority of self-reported questionnaires showed high or moderate correlation with medication adherence using a monitoring device and are suitable for measuring patient-reported adherence [28].

Previous studies have found that patients who have been educated till the primary level showed significantly low adherence to medication [14,26]. The association may be due to the relationship between education and other variables. For example, the educational qualification could determine a patient’s trust in physician and could further differ according to different levels of education [29]. However, in this study we did not find any association between low medication adherence and education level.

Low socioeconomic status is a significant factor for poor adherence to medication among diabetic patients [15,30,31]. A recent study in Ethiopia showed that being male and having poor wealth status was associated with low medication adherence [32]. Align to our findings different studies have reported women to be more adherent than men [33,34]. Our results were in line with the trend that women were more adherent, but we ruled out the association between low socioeconomic status and poor adherence. Several other studies have suggested that medication adherence has no relationship with demographic variables, such as gender, age, and socioeconomic status [35,36].

In the unadjusted analysis, we found a significant association between low medication adherence in diabetic individuals and the number of medications they consume which is contrary to the findings of other studies [8,26]. However, the association between the number of medications consumed and non-adherence in our bivariate analysis became less significant when we incorporated it into our multivariate analysis. A review paper on medication adherence for different diseases supports this result; it had mixed findings regarding the relationship between the number of medications taken and poor adherence to them [29].

In this study, patients who consumed fewer fruits and vegetables and had ocular disorders and diabetic ulcers showed a significant association with low medication adherence. A previous study on anti-hyperglycemic drugs found that patients with satisfactory refill adherence also had higher adherence to cardiovascular drugs than non-adherent patients [37], indicating that less-adherent diabetic patients may be at a significantly higher risk of suboptimal cardio-metabolic control and poor clinical outcomes [38]. Patients with a family history of diabetes were not significantly associated with low medication adherence in this study, which is similar to the findings of a previous study conducted in Pakistan [39]. Reportedly, the family members of diabetes patients are more knowledgeable about diabetes but they perform more non-supportive behaviors and lead to patients being less adherent to their medication [40].

There are a few limitations to this study. First, we primarily included subjects residing in urban areas who were outpatients at clinics, hospitals, and diagnostic centers within Chittagong city and had access to specialized care and education on diabetes management protocols. However, diabetic individuals in rural areas are deprived of expert medical advice. This means that although our sample size was large enough to evaluate the expected differences and associations between variables, our findings cannot be generalized to the entire diabetic population of Bangladesh. Second, we were unable to collect data on several contributing factors, such as health literacy, food frequency, and pathophysiological factors that could be relevant to medication adherence. Third, our patients may have overestimated their medication adherence in their self-reported assessments, but we could not validate our results with more accurate measurements of adherence. Regardless of these limitations, this is a pioneering study in Bangladesh that provides a novel country-specific analysis of medication adherence among patients with type 2 diabetes using a standardized self-reported questionnaire.

## Conclusion

Despite the public health efforts being made to effectively manage diabetes among the population of Bangladesh, increasing medication adherence is still a key challenge among patients with type 2 diabetes in southern Bangladesh. The factors we identified to be associated with low medication adherence among diabetic individuals included male gender, family income of less than 233 USD, diabetic ulcers, and lower consumption of fruits and vegetables (less than 3 times a day). Medication adherence may be even lower in remote areas of the country where access to care is limited. Our findings will help physicians and public health workers identify further factors that cause poor adherence and design innovative interventions to address these and eventually improve medication adherence in Bangladesh.

## Paper context

Low adherence to anti-diabetes medication precludes poor glycemic control in individuals with type 2 diabetes. Almost 50% of our study population (patients with type 2 diabetes from five health facilities in Bangladesh) had low medication adherence despite receiving optimal care and medical advice. Male gender, low family income, and diabetic ulcers were the most influential factors for a low level of medication adherence. Addressing these issues will allow physicians to implement more competent treatment methods and ensure a better quality of life for their patients.
